# A Template-Free, Ultra-Adsorbing, High Surface Area Carbonate Nanostructure

**DOI:** 10.1371/journal.pone.0068486

**Published:** 2013-07-17

**Authors:** Johan Forsgren, Sara Frykstrand, Kathryn Grandfield, Albert Mihranyan, Maria Strømme

**Affiliations:** 1 Division for Nanotechnology and Functional Materials, Department of Engineering Sciences, The Ångström Laboratory, Uppsala University, Uppsala, Sweden; 2 Division for Applied Materials Science, Department of Engineering Sciences, The Ångström Laboratory, Uppsala University, Uppsala, Sweden; Massey University, New Zealand

## Abstract

We report the template-free, low-temperature synthesis of a stable, amorphous, and anhydrous magnesium carbonate nanostructure with pore sizes below 6 nm and a specific surface area of ∼ 800 m^2^ g^−1^, substantially surpassing the surface area of all previously described alkali earth metal carbonates. The moisture sorption of the novel nanostructure is featured by a unique set of properties including an adsorption capacity ∼50% larger than that of the hygroscopic zeolite-Y at low relative humidities and with the ability to retain more than 75% of the adsorbed water when the humidity is decreased from 95% to 5% at room temperature. These properties can be regenerated by heat treatment at temperatures below 100°C.The structure is foreseen to become useful in applications such as humidity control, as industrial adsorbents and filters, in drug delivery and catalysis.

## Introduction

Nanotechnology is starting to influence most scientific areas and this key enabling technology is foreseen to significantly impact all materials science dependent industries during the coming decades [1]–[4]. The interest in high surface area nanostructured materials from 1990 onwards has increased exponentially for all classes of porous materials and at the beginning of 2013, according to the ISI Web of Knowledge, there were in total about 60,500 records on zeolites, 20,500 records for mesoporous silica and 13,100 records on metal organic framework (MOF) materials, whereas before 1990 these numbers were insignificant. The most common way to produce high surface area materials with micro-mesoporous structures, i.e., pores with diameters below 50 nm, is by using soft templates and building around them a more rigid structure after which the template is eluted with a solvent or burnt away to produce the rigid porous material.

In the current work we will show that it is possible, at low temperatures and without the use of templates, to synthesize a unique high surface area nanostructure with a well-defined pore-size distribution of sub 6 nm pores of a widely used, non-toxic and GRAS (generally-recognised-as-safe)-listed material that is already included in the FDA Inactive Ingredients Database [5]; viz. magnesium carbonate.

Magnesium is the eighth most abundant element in the earth’s crust and essential to most living species. It can form several structures of hydrated carbonates such as nesquehonite (MgCO_3_·3H_2_O), and lansfordite (MgCO_3_·5H_2_O), a number of basic carbonates such as hydromagnesite (4 MgCO_3_·Mg(OH)_2_·4 H_2_O), and dypingite (4 MgCO_3_·Mg(OH)_2_·5 H_2_O), as well as the anhydrous and rarely encountered magnesite (MgCO_3_) [5]. In contrast to other alkali earth metal carbonates, chemists have found anhydrous magnesium carbonate difficult to produce, particularly at low temperatures. Above 100°C, magnesite (crystalline MgCO_3_) can be obtained from Mg(HCO_3_)_2_ solutions by precipitation. However, at lower temperatures, hydrated magnesium carbonates tend to form, giving rise to what has been referred to as “*the magnesite problem*” [6].Yet, not only chemists have been intrigued by magnesium carbonates. Although abundant in nature, where crystalline forms exist as traces in most geological structures, pure magnesium carbonate is seldom found on its own in larger deposits, a fact that has puzzled geologist for more than a century [7].

In 1908, Neuberg and Rewald tried to synthesise magnesite in alcohol suspensions of MgO [8]. However, it was concluded that MgCO_3_ cannot be obtained by passing CO_2_ gas through such suspensions due to the more likely formation of magnesium dimethyl carbonate (Mg(OCOOCH_3_)_2_). Subsequent studies by Kurov in 1961 [9] and Buzágh in 1926 [10] only reiterated the assumption that MgO preferentially forms complex dimethyl carbonates when reacted with CO_2_ in methanol. A further overview of early works is provided in detail in Text S1 and Figure S1.

Yet, by changing the synthesis conditions in comparison to what has been described earlier, we here report the successful formation of a magnesium carbonate, hereafter referred to as *Upsalite*, in a reaction between MgO, methanol and CO_2_ resulting in an anhydrous, micro-mesoporous and large surface area structure. We further show that the moisture sorption of the material is featured by a unique set of properties including an adsorption capacity ∼50% larger than that of the hygroscopic zeolite-Y at low relative humidities and with the ability to retain more than 75% of the adsorbed water when the humidity is decreased from 95% to 5% at room temperature. The humid material is easily regenerated to regain its moisture sorption characteristic upon storage at only 95°C.

## Results and Discussion

**Figure 1:**
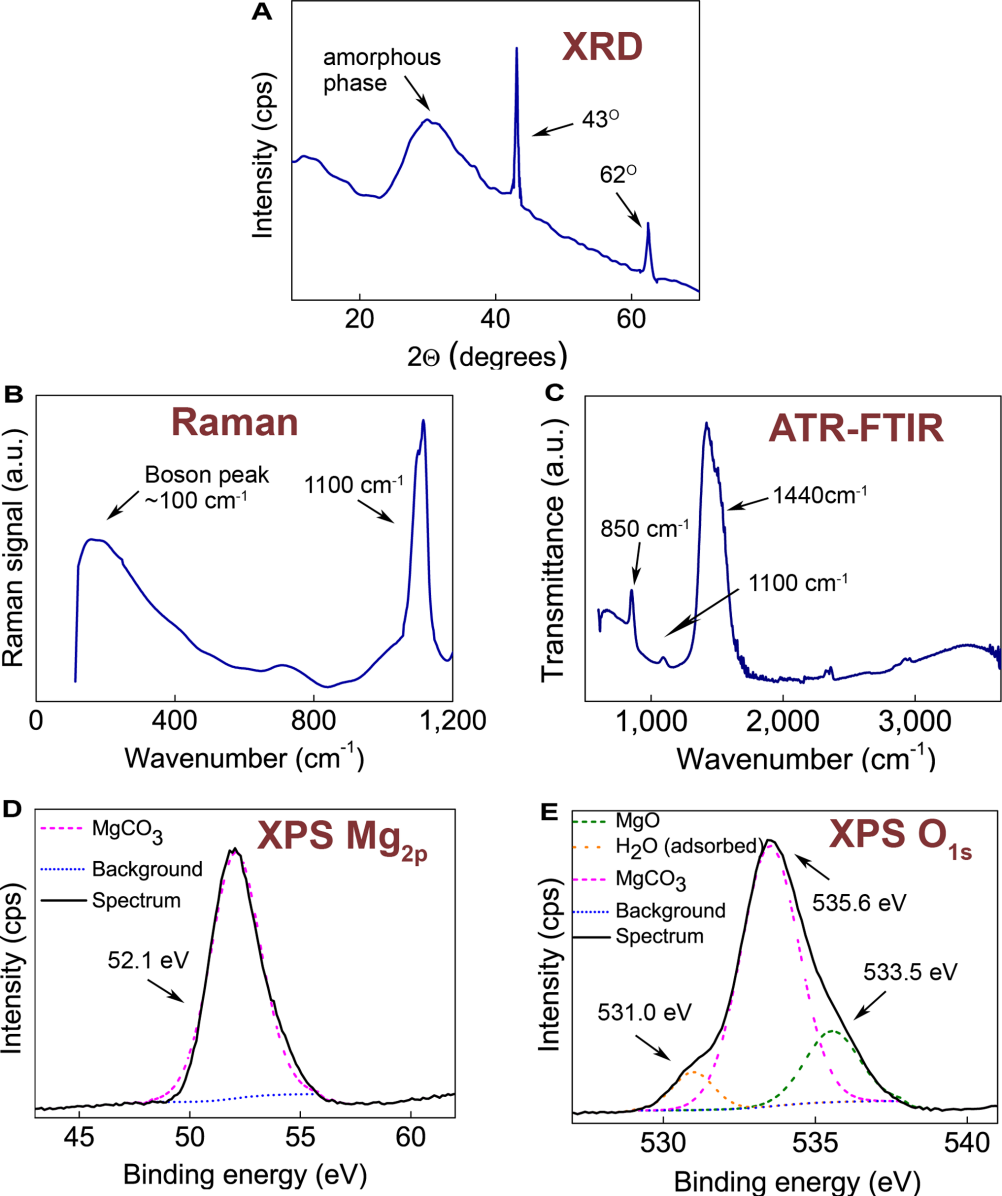
Characterisation of as-synthesised Upsalite and schematic description of the synthesis steps. **a)** XRD pattern. The halo at 2 θ∼30° indicates the presence of at least one amorphous phase and the sharp peaks at higher scattering angles pertain to crystalline MgO. **b)** Raman spectrum. The band observed at ∼1100 cm^−1^ stems from vibration of the CO_3_ group and the halo cantered at 100 cm^−1^ is a Boson peak. **c)** FTIR spectrum. The three visible absorption bands (1440 cm^−1^, 1100 cm^−1^ and 850 cm^−1^) are all due to vibrations of the CO_3_ group. **d)** and **e)** XPS Mg_2p_ and O_1s_ peaks. The Mg_2p_ peak at 52.1 eV and the O_1s_ peak at 533.5 eV stem from MgCO_3_, the O_1s_ peak at 531.0 eV from MgO and the O_1s_ peak at 535.6 eV from surface adsorbed water. The solid lines represent the measured spectrum. The coloured lines are calculated using the CasaXPS software and represent the fitted curves (obtained using Gaussian-Lorentzian functions) and the subtracted background (obtained using a Shirley function).

The synthesis is carried out well below 100°C, while previously reported amorphous structures of magnesium carbonate have been formed at higher temperatures by thermal decomposition of hydrated magnesium carbonates [11]–[14] or of a double salt of magnesium ammonium carbonate [15]. In the current work, CO_2_ is not bubbled through the methanolic suspension, instead the reaction vessel is pressurised with CO_2_ to moderate relative pressures (1–3 bar). Initially, the temperature is kept at 50°C in order to facilitate a reaction between MgO and methanol, and after ∼ 3 h the temperature is decreased to room temperature. This results in formation of a rigid gel in the reaction vessel after ∼ 4 days. When dried in air at 70°C, the gel solidifies and collapses into a white and coarse powder that is primarily X-ray amorphous with traces of unreacted and crystalline MgO, see XRD pattern in Figure 1a. The sharp peaks at 2 θ equal to 43° and 62° originate from the unreacted MgO [16], while the halo peak between 2 θ values of 25° and 40° is indicative of at least one amorphous phase.

Raman spectroscopy reveals that the powder indeed is composed of a carbonate (Figure 1b), where the band at ∼1100 cm^−1^ corresponds to vibration of the carbonate group [17]. Moreover, a broad halo, or the so-called Boson peak, with a maximum at ∼100 cm^−1^, is further witness to the amorphous character of the powder [18].

When examined with Fourier transform infrared spectroscopy (FTIR, Figure 1c) the material displayed absorption bands at ∼1440 cm^−1^, ∼1100 cm^−1^ and ∼850 cm^−1^, which all correspond to the carbonate group [19]. No water of crystallisation is visible in this spectrum [13], [19]. The anhydrous character of the bulk material is further confirmed by Thermal Gravimetric Analysis (TGA) (see Figure S2).

X-ray photoelectron spectroscopy (XPS) confirms the anhydrous nature of the MgCO_3_. Energy resolved spectra were recorded for the Mg_2p_ and O_1s_ peaks (Figure 1d,e) which were found to be positioned at 52.1 eV and 533.5 eV, respectively, which is indicative of MgCO_3_ [20]. Further, the O_1s_ peak does not contain any components for crystal water which, expectedly, would have appeared at 533–533.5 eV [21]. The shoulder seen at 535.6 eV is located between the binding energies for liquid water (539 eV) and ice (533 eV) [22] and is, therefore, representative of surface adsorbed water as previously described for adsorbed water on carbon fibres [23], [24]. The shoulder seen at 531.0 eV shows the presence of MgO in the powder. No presence of Mg(OH)_2_ was observed in the bulk, which would have resulted in a peak at 532.4 eV [20].

Having proved the formation of amorphous anhydrous MgCO_3_ by XRD, Raman, FTIR, and XPS, we postulate the following simplified route of synthesis, based on the presence of HOMgOCH_3_ as an intermediate (as confirmed with FTIR in Figure S3) and the necessity of heat treatment in the last synthesis step:
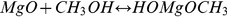
(1a)

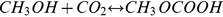
(1b)

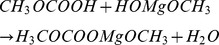
(1c)

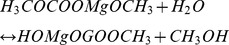
(1d)


The presence of a hydroxyl group in the vicinity of the methoxy group in HOMgOCOOCH_3_ makes the compound labile and will therefore favour internal transition to a solvate MgCO_3_·CH_3_OH and loss of methanol upon heating to 70°C:
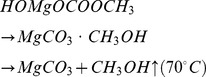
(1e)


**Figure 2:**
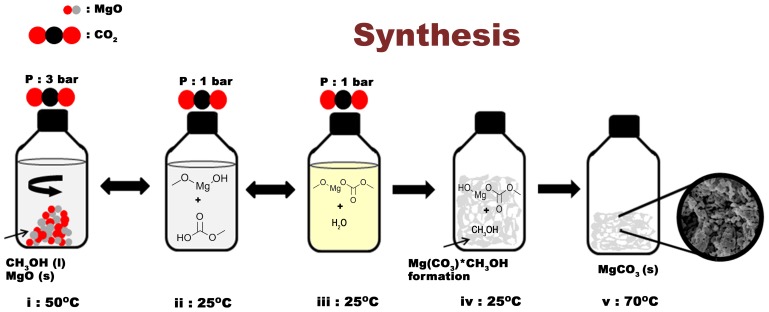
Synthesis of Upsalite. **i)** In the first step MgO (s) is mixed with methanol under 3 bar CO_2_ pressure at 50°C. **ii)** After 2.5 h the HOMgOCH_3_ is formed in the solution, the pressure is lowered to 1 bar and the heating is turned off. At the same time the methanol reacts with the CO_2_ and forms CH_3_OCOOH (methyl hemicarbonic acid). **iii)** HOMgOCH_3_ reacts with CH_3_OCOOH and forms water and H_3_COCOOMgOCH_3_ (methyl esther of magnesium methyl carbonate). At this point the solution changes colour from white to light yellow. **iv)** H_3_COCOOMgOCH_3_ reacts with the water formed in step iii) and forms HOMgOCOOCH_3_ (or MgCO_3_·CH_3_OH) which upon **v)** heating at 70°C releases CH_3_OH and forms MgCO_3_.

As it is evident from the above cascade scheme, the postulated reaction of MgCO_3_ formation goes through several steps, some of which are equilibrium reactions, namely 1a and 1b. A schematic description of the reaction steps is presented in Figure 2.

**Figure 3:**
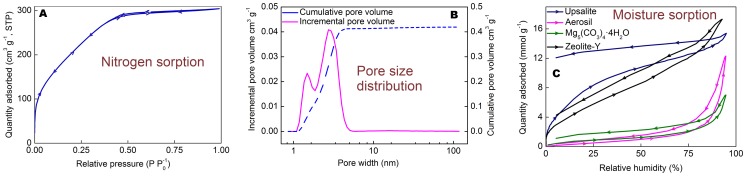
Sorption isotherms and DFT-based pore size distribution for Upsalite. **a)** N_2_ sorption isotherm at 77 K. **b)** Incremental pore volume (violet) and cumulative pore volume (blue) obtained from N_2_ sorption isotherm. **c)** Moisture sorption isotherm at room temperature for Upsalite (blue), Mg_5_(CO_3_)_4_(OH)_2_·4H_2_O (green), Aerosil (red) and Zeolite Y (black). The arrows indicate the direction of the pressure change.

In order to analyse the pore structure and water sorption capacity of Upsalite, N_2_ and H_2_O vapour sorption analyses were performed. Figure 3a shows the N_2_ sorption isotherm for Upsalite, which exhibits a typical Type 1 shape according to the IUPAC classification [25]. The SSA of 800 m^2^ g^−1^ for the material (Table 1) was derived from such isotherms according to the Brunauer-Emmet-Teller (BET) equation [26], substantially surpassing the SSA of all previously described alkali earth metal carbonates, where crystalline forms of magnesium carbonates typically have a SSA of 4–18 m^2^ g^−1^ [5]. This high SSA places Upsalite in the exclusive class of high surface area nanomaterials including mesoporous silica, zeolites, MOFs, and carbon nanotubes.

**Table 1:** Structural and chemical characteristics of Upsalite obtained from N_2_ and H_2_O vapour sorption isotherms.

Adsorbate	N_2_	H_2_O
SSA^[a]^ (m^2^/g)	800.0±3.6	–
Total pore volume^[b]^ (cm^3^/g)	0.47	–
*w_0_*, limiting micropore volume^[c]^ (cm^3^/g)	0.280±0.001	0.160±0.010
Equivalent surface area in micropores^[c]^ (m^2^/g)	549	478
Characteristic energy of adsorption^[c]^ (kJ/mol)	11.4	41.0
Modal equivalent pore width^[c]^ (nm)	1.75	1.09
Correlation coefficient of fit^[c]^	0.999	0.977
^[a]^According to the BET equation		
^[b]^Single point adsorption at P/P_0_≈1		
^[c]^According to the Dubinin-Astakhov equation		

Figure 3c displays the H_2_O vapour sorption isotherm for Upsalite and, based on the large amount of H_2_O adsorbed at low RHs, it is evident that the material is highly hydrophilic [27]. The limited desorption of moisture from the material when the vapour pressure is reduced from 95% is further proof of the strong interaction between water molecules and the material. It should, however, be noted that no signs of hydrate formation in the material are seen using XRD after the sorption isotherm is completed, and that the sorption isotherm can be repeated with undistinguishable results after heat treatment at moderate temperatures (95°C) under vacuum. This contrasts to the regeneration of moisture sorption properties of, e.g., Zeolites typically requiring heat treatments at temperatures between 150°C and ∼600°C.

Further, both the N_2_ and the H_2_O vapour sorption isotherms were analysed in order to establish the microporous properties of the material according to the Dubinin-Astakhov (D-A) model [28], see Table 1. The hydrophilic nature of the material is further reflected in the greater characteristic energy for adsorption of H_2_O compared to N_2_. The discrepancy in the limiting micropore volume (*w_0_*) – in which the value obtained from the N_2_ sorption isotherm is to be regarded as the “true” value – and modal equivalent pore size obtained from the two sorption isotherms is most likely due to site-specific interaction between the H_2_O species and the material, not only in the micropores but also on the exterior of the material and in pores larger than 2 nm [29].

Figure 3b shows the incremental and cumulative pore volume obtained through density functional theory (DFT) calculations on the N_2_ sorption isotherm. From these calculations it infers that about 98% of the pore volume is inherent to pores with a diameter smaller than 6 nm, while the remaining pore volume is made up of pores with a broad size distribution between 8 and 80 nm centred at ∼16 nm.

**Figure 4:**
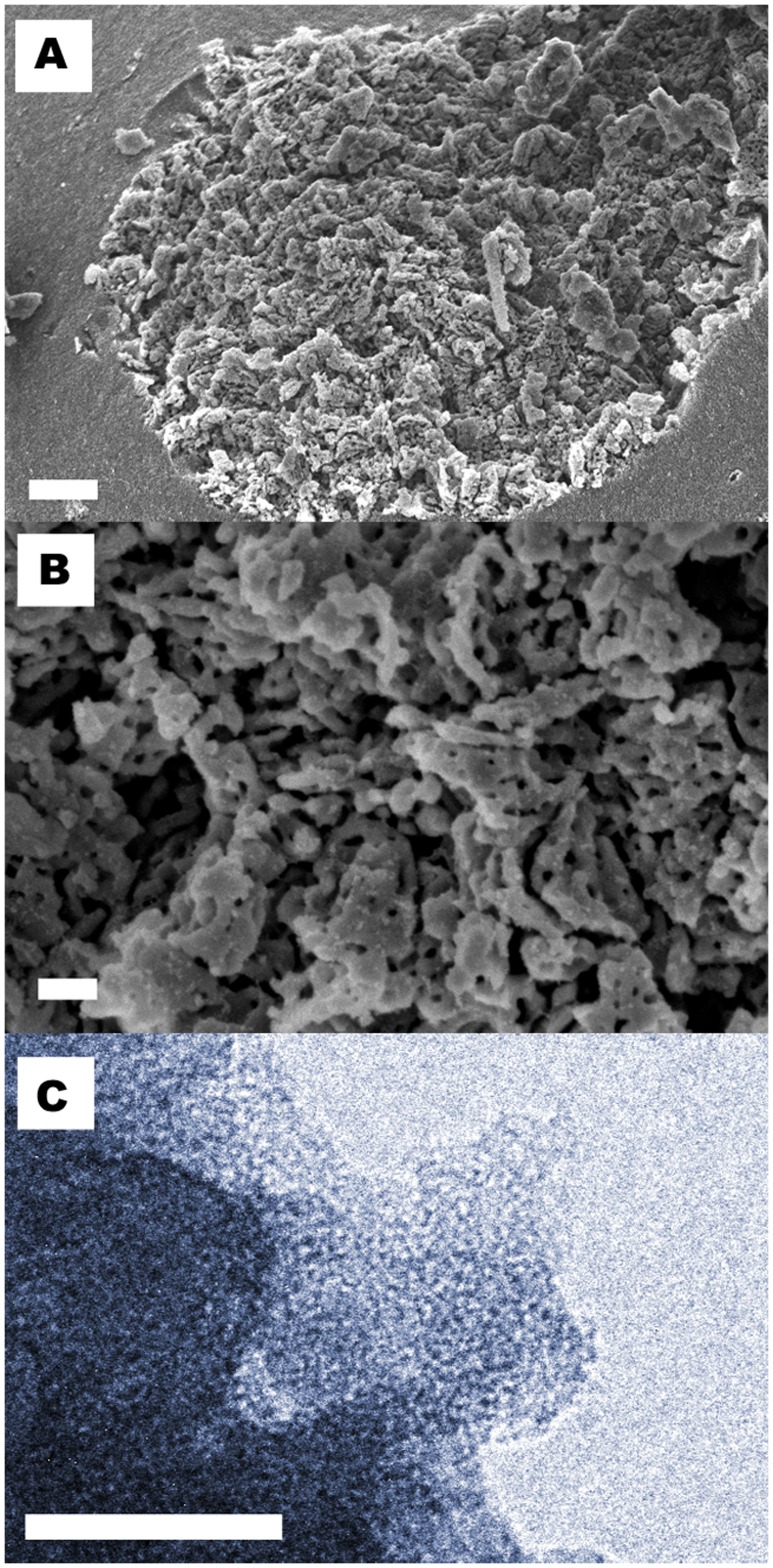
Electron microscopy images of Upsalite. **a)** SEM micrograph of Upsalite. Scale bar, 1 µm. **b)** Higher magnification SEM of a region in a) clearly showing the textural porosity of the material. Scale bar, 200 nm. **c)** Representative TEM image of Upsalite showing contrast consistent with a porous material. The image is recorded with under-focused conditions to enhance the contrast from the pores. Scale bar, 50 nm.

When examined with scanning electron microscopy (SEM), these pores in the larger size range are clearly visible (Figure 4a–b). Furthermore, the highly porous nature of the material is evident from the scanning transmission electron microscopy (STEM) tomography work available in the Supporting Information (see Video S1 and S2 and Figure S4; S4.1). Such three-dimensional reconstructions allow for visualisation of the internal pore structure of the material via a series of slices through the volume. Measurements from these slices, which essentially represent cross-sections, confirm that pore widths are consistently 16 nm, while pore heights vary between 8 and 50 nm. However, these larger pore networks that are visible with SEM and STEM are not noted throughout the entire material, which is consistent with the limited contribution from these pores to the total pore volume as determined by DFT. In the transmission electron microscopy (TEM) image in Figure 4c the smaller pores, dominating the contribution to the total pore volume sensed by nitrogen sorption, can be distinguished. An enlargement of pores was found to take place under the electron beam where the sample was unstable for long periods of time. This enlargement is most likely due to remaining organic groups leaving the sample. A representative image recorded after a longer period (∼ 1 min) under the electron beam is shown in Figure S4 (S4.2).

The water sorption capacity of the material is interesting from an industrial and technological point of view and it is, hence, compared to three commercially available desiccants, namely fumed silica (SSA: 196 m^2^ g^−1^), hydromagnesite (SSA: 38 m^2^ g^−1^) and the microporous Zeolite Y (SSA: 600 m^2^ g^−1^, silica/alumina ratio 5.2∶1), see Figure 3c. For comparison, all samples were degassed at 95°C under vacuum for 10 h prior to analysis. The H_2_O vapour adsorption isotherm for Upsalite displays similarities with the hydrophilic zeolite at very low RHs (<1%) and shows an even higher adsorption capacity compared to the zeolite at RHs between 1 and 60%. This behaviour contrasts largely to that of the other two non-porous materials, i.e. fumed silica and hydromagnesite, which mainly adsorb H_2_O at RH >60%.

Amorphous magnesium carbonates produced by high temperature thermal decomposition of hydromagnesite, nesquehonite, or magnesium ammonium carbonate double salt have previously been reported to be unstable upon hydration [15], [30]. In particular, instability of the hydromagnesite decomposed material was evident by a weakening of the carbonate bond [30]. Such weakening was observed by a shift towards lower temperatures, as well as by the development of a shoulder and a split into two or more peaks, of the carbonate decomposition peak located above 350°C in differential TGA (dTGA) spectra [30]. In this respect, Upsalite appears to remain stable upon hydration. After 11 weeks of storage at RT and 100% RH no peak split, shoulders or movement of the carbonate decomposition peak towards lower temperatures is observed in dTGA spectra (see Figure S5). In fact, the decomposition peak for the carbonate bond is shifted towards higher temperatures as compared to the as-synthesized material (Figure S2 and S5), indicating a strengthening of the carbonate bond.

### Conclusions

We report herein the template-free formation of a stable, amorphous magnesium carbonate nanostructure formed at low temperature in a methanol solution of MgO with CO_2_. The obtained magnesium carbonate is featured with a unique structure of pores almost exclusively in the sub 6 nm size range and extraordinarily high surface area, which has never been reported before, neither for natural nor synthetic magnesium carbonates. The material described in this work is further featured with extraordinary water sorption properties that can be regenerated at temperatures below 100°C. The material is foreseen to find its use in a number of applications including humidity control and delivery systems for therapeutic or volatile agents.

## Materials and Methods

### Synthesis

In the current work 4 g magnesium MgO powder was placed in a glass bottle together with 60 ml methanol and a stirring magnet. The solution was put under 3 bar CO_2_ pressure and heated to 50°C. After approximately 4 hours the mixture was allowed to cool to RT and the carbon dioxide pressure was lowered to 1 bar, and the reaction continued until a gel had formed. When a gel was obtained, the carbon dioxide pressure was removed and the gel was allowed to solidify and dry at ∼70°C during 3 days. A schematic description of the synthesis is found in Fig. 2.

### Characterisation

#### X-ray diffraction

XRD analysis was performed with a Siemens/Bruker D5000 instrument using Cu-K_α_ radiation. Samples were ground and put on a silicon sample holder with zero background prior to analysis. The instrument was set to operate at 45 kV and 40 mA.

#### Raman spectroscopy

A Reinshaw Ramanscope was used for the Raman studies. The Raman instrument was calibrated with a silicon wafer using the band at 521 cm^−1^ prior to the studies. A 524 nm argon-ion laser with a 10 µm spot size was used for analysis.

#### Fourier transform infrared spectroscopy

The FTIR studies were performed on a Bruker IFS 66v/S spectrometer using an Attenuated Total Reflectance (ATR) sample holder from SENSIR. 50 scans were signal-averaged in each spectrum and the resolution was 4 cm^−1^. Before the measurement a background scan was recorded and thereafter subtracted from the spectrum for the sample.

#### X-ray photoelectron spectroscopy

The XPS experiments were conducted on a Phi Quantum 200 Scanning ESCA microprobe instrument. Prior to analysis, the samples were sputter cleaned using argon ions for 10 min at 200 V to remove surface adsorbed contaminations. A full spectrum was recorded together with energy resolved spectra for Mg_2p_ and O_1s_. During the acquisition, an electron beam of 20 µA was used together with argon ions to neutralise the non-conducting sample. The peak fittings were made with CasaXPS software, the curves were fitted using Gaussian-Lorentzian functions and the background was subtracted using a Shirley function. The spectra were calibrated against the O_1s_ peak for magnesium oxide (531.0 eV) instead of the C_1s_ peak at 285.0 eV for adventitious carbon, which otherwise is commonly used as a reference. However, in the case of MgO, the binding energy for adventitious carbon is not reliable as reference since hydrocarbons interact with magnesium oxide in a way that shifts the C_1s_ peak randomly making it unsuitable as a reference. Therefore, the O_1s_ peak for MgO (531.0 eV) is instead proposed to be used as an internal reference [31]. The presence of magnesium oxide in the samples was confirmed by XRD analysis prior to the XPS study.

#### N_2_ sorption analysis

Gas sorption measurements were carried out with N_2_ at 77 K using an ASAP 2020 from Micromeritics. The samples were degassed at 95°C under vacuum for 10 h prior to analysis. The SSA was determined by applying the BET equation [26] to the relative pressure range of 0.05–0.30 for the adsorption branch of the isotherm. The D-A equation was employed on the appropriate pressure region for adsorption in micropores. The BET and D-A calculations were performed with the ASAP 2020 V3.04 software from Micromeritics delivered together with the analysis equipment. The pore size distribution was determined using *DFT analysis* carried out with the DFT Plus software from Micrometrics using the model for N_2_ at 77K for slit-shape geometry with low regularisation (λ = 0.005). The standard deviation of the DFT fit was 2.037 cm^3^/g.

#### Scanning electron microscopy

For the SEM analyses, a Leo 1550 instrument from Zeiss equipped with an in-lens detector was used. Prior to the studies, the samples were cooled with liquid N_2_, crushed and put on a stub holder with double-sided carbon tape. As a last step prior to analysis the sample was sputter coated with a thin layer of gold/palladium.

#### Thermal gravimetric analysis

TGA analysis was carried out under a flow of air in an inert alumina cup with sample sizes of approximately 15 mg. The samples were heated from RT to 700°C with a heating ramp of 10°C min^−1^ using a Thermogravimetric analyser from Mettler Toledo, model TGA/SDTA851e.

#### Water vapour sorption

An ASAP 2020 instrument from Micromeritics was used for the water sorption studies. Prior to analysis the samples were degassed at 95°C under vacuum for 10 h. The D-A equation was employed on the appropriate pressure region for adsorption in micropores. The affinity coefficient (*β*) for water was set to 0.2 in the D-A calculations, which has been shown to be an appropriate value for analysis of polar surfaces [32].

#### Scanning transmission electron microscopy and electron tomography

Scanning transmission electron microscopy (STEM) samples were prepared by dispersing the powder in ethanol and placing 20 µl on a Quantifoil® TEM grid. Experiments were performed on an FEI Tecnai F20 (FEI Company, The Netherlands) operated at 200 kV. Images were recorded on a high-angle annular dark-field detector (HAADF). The Dual-Axis Tomography Holder Model 2040 (Fischione Instruments, PA, USA) was used in a linear tilt scheme to acquire a single-axis tilt-series with image acquisition increments of 2°. Automated focusing, image shifting, and acquisition of HAADF STEM images over an angular range of ±62° were achieved using the Explore3D software (FEI Company, The Netherlands). The 3D reconstructions were computed using a simultaneous iterative reconstruction technique, with 20 iterations, in Inspect3D (FEI Company, The Netherlands). Models for 3D visualisation were created in Amira Resolve RT FEI (Visage Imaging Inc., USA).

#### Transmission Electron Microscopy (TEM)

HRTEM images were taken with a JEOL-3010 microscope, operating at 300 kV (Cs 0.6 mm, resolution 1.7 Å). Images were recorded using a CCD camera (model Keen View, SIS analysis, size 1024×1024, pixel size 23.5×23.5 µm) at 30 000–100 000× magnification using low-dose conditions on as-crushed samples.

## Supporting Information

Figure S1 **X-ray pattern of the material obtained when water was deliberately added to the synthesis.** All peaks in the pattern corresponds to nesquehonite, Mg(HCO_3_)(OH)·2 H_2_O, (PDF# 00-020-0669). No signs of residual MgO can be detected in the pattern.
(DOCX)

Figure S2 **Thermal Gravimetric Analysis of Upsalite. TGA and dTGA curves for Upsalite.**
(DOCX)

Figure S3 **FTIR spectrum for the** ***in-situ*** **sample collected from the reaction vessel after 3 hours of reaction, together with a reference sample.**
(DOCX)

Figure S4 **STEM Tomography and TEM. Internal pore structure from electron tomography.** Slices through two electron tomographic reconstructions of the Upsalite material. Essentially representing an internal cross-section, pore size measurements from these volumetric slices indicate pore sizes with one dimension measuring on average 16 nm, and the other varying between 50 nm and 8 nm (S4.1). TEM image showing Upsalite sample after a long period (∼1 min) under the electron beam (S4.2).
(DOCX)

Figure S5 **Differential Thermal Gravimetric Analysis of Upsalite stored at 100% humidity and RT for different time periods.**
(DOCX)

Text S1 **Overview of earlier work.**
(DOCX)

Video S1 **Electron tomographic tilt-series.** The as-acquired electron tomographic tilt-series of Upsalite comprised of HAADF STEM images taken over ±62°.
(ZIP)

Video S2 **Electron tomographic reconstruction.** Volumetric slices through the reconstructed volume, followed by a three-dimensional rendering, reveal the internal pore structure of Upsalite.
(MPG)
