# Correction: A Template-Free, Ultra-Adsorbing, High Surface Area Carbonate Nanostructure

**DOI:** 10.1371/annotation/0878101b-450f-44a9-abd5-b2434957a698

**Published:** 2014-01-16

**Authors:** Johan Forsgren, Sara Frykstrand, Kathryn Grandfield, Albert Mihranyan, Maria Strømme

During production, an error was introduced into Equation 1d. The correct version is H3COCOOMgOCH3+H2O↔HOMgOCOOCH3+CH3OH.

