# Supplementary material for: A Template-Free, Ultra-Adsorbing, High Surface Area Carbonate Nanostructure
Source: PLoS One. 2013 Jul 17;8(7):e68486. doi: 10.1371/journal.pone.0068486 (PMC3714275; doi:10.1371/journal.pone.0068486)
Supplement: Figure S1 — X-ray pattern of the material obtained when water was deliberately added to the synthesis. All peaks in the pattern corresponds to nesquehonite, Mg(HCO3)(OH)·2 H2O, (PDF# 00-020-0669). No signs of residual MgO can be detected in the pattern. (DOCX) [file pone.0068486.s001.docx]

SUPPORTING FIGURE S1 for

A template-free, ultra-adsorbing, high surface area carbonate nanostructure

Johan Forsgren, Sara Frykstrand, Kathryn Grandfield, Albert Mihranyan, and Maria Strømme


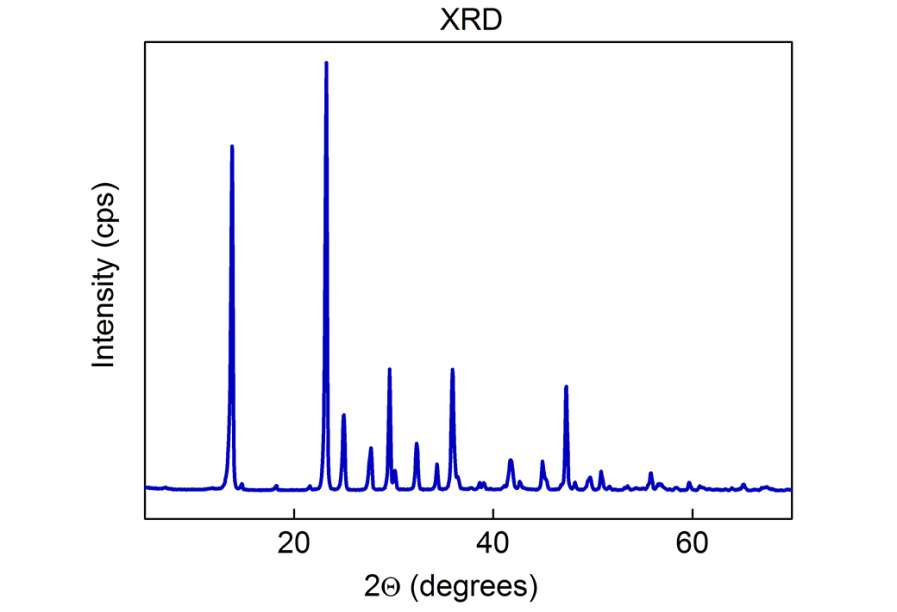


**Figure S1.** X-ray pattern of the material obtained when water was deliberately added to the synthesis. All peaks in the pattern corresponds to nesquehonite, Mg(HCO_3_)(OH)·2H_2_O, (PDF# 00-020-0669). No signs of residual MgO can be detected in the pattern.
