# Supplementary material for: A Template-Free, Ultra-Adsorbing, High Surface Area Carbonate Nanostructure
Source: PLoS One. 2013 Jul 17;8(7):e68486. doi: 10.1371/journal.pone.0068486 (PMC3714275; doi:10.1371/journal.pone.0068486)
Supplement: Figure S2 — Thermal Gravimetric Analysis of Upsalite. TGA and dTGA curves for Upsalite. (DOCX) [file pone.0068486.s002.docx]

SUPPORTING FIGURE S2 for

A template-free, ultra-adsorbing, high surface area carbonate nanostructure

Johan Forsgren, Sara Frykstrand, Kathryn Grandfield, Albert Mihranyan, and Maria Strømme

**S2. TG analysis of Upsalite**

TG analysis was used to investigate the relative proportions of the constituents in the material. The TGA curve can be seen in Fig. S2 where a rapid weight loss, corresponding to the decomposition of the magnesium carbonate to MgO,^1^ is observed at ~390 °C in the dTGA curve. Based on the molar fraction of released CO_2_ during decomposition at ~450 °C, the relative weight ratio between residual MgO and magnesium carbonate in Upsalite was found to be 1:6 and the corresponding molar ratio 1:2.8. The TG analysis also shows a major weight loss with maximum at 160 °C that is due to the loss of physisorbed water and possible remaining organic groups. Chemisorbed water does not evaporate until the temperature reaches 300 °C,[^43^](#_ENREF_43) thus TGA further establishes the anhydrous character of Upsalite.


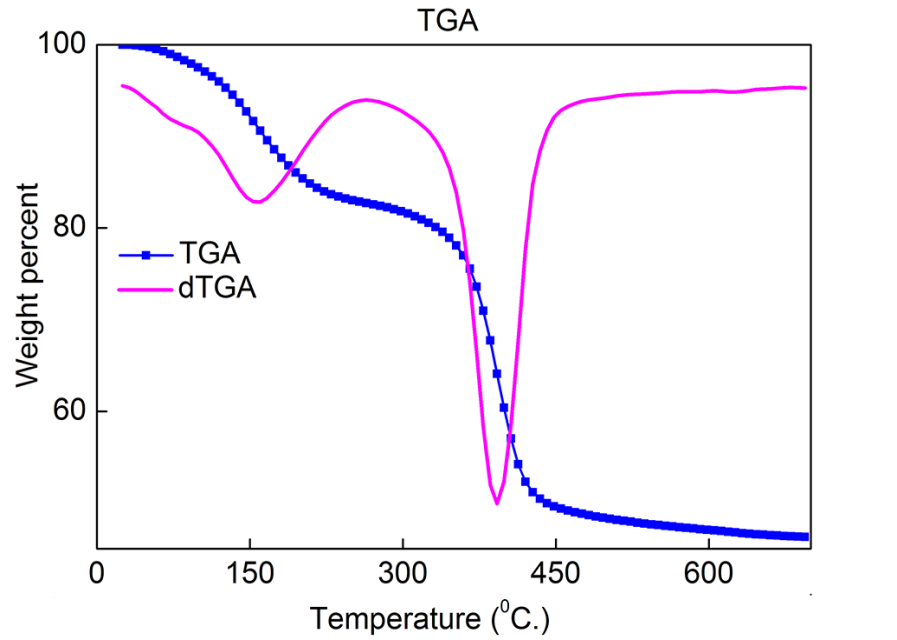


**Figure S2.** Thermal Gravimetric Analysis of Upsalite. TGA (blue) and dTGA (pink) curves for Upsalite.

**SUPPORTING REFERENCES**

1 Botha, A.; Strydom, C. A. Dta and Ft-Ir Analysis of the Rehydration of Basic Magnesium Carbonate. *J. Therm. Anal. Calorim.* **2003,** *71*, 987-995.
