# Supplementary material for: A Template-Free, Ultra-Adsorbing, High Surface Area Carbonate Nanostructure
Source: PLoS One. 2013 Jul 17;8(7):e68486. doi: 10.1371/journal.pone.0068486 (PMC3714275; doi:10.1371/journal.pone.0068486)
Supplement: Figure S3 — FTIR spectrum for the in-situ sample collected from the reaction vessel after 3 hours of reaction, together with a reference sample. (DOCX) [file pone.0068486.s003.docx]

SUPPORTING FIGURE S3 for

A template-free, ultra-adsorbing, high surface area carbonate nanostructure

Johan Forsgren, Sara Frykstrand, Kathryn Grandfield, Albert Mihranyan, and Maria Strømme

**S3. FTIR spectrum for the intermediate product formed during the first reaction step**

**
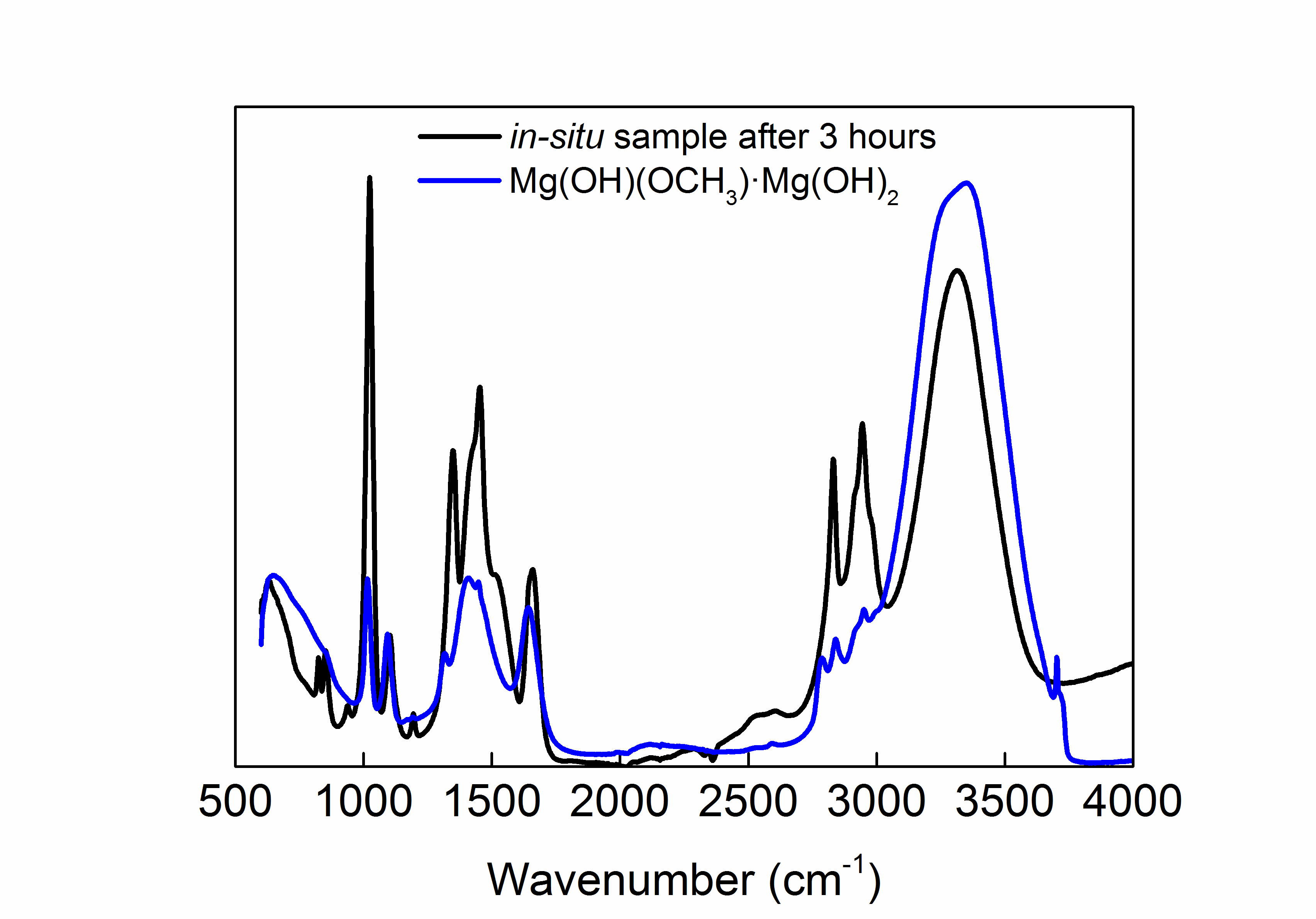
**

**Figure S3.** FTIR spectrum for the *in-situ* sample collected from the reaction vessel after 3 hours of reaction, together with a reference sample.

A small sample was withdrawn from the liquid in the reaction vessel after 3 h of reaction. The obtained sample was analysed with FTIR in order to establish its constituents, see Fig. S3. A reference sample consisting of Mg(OH)(OCH_3_)·Mg(OH)_2_ was prepared by hydrolysis of Mg(OCH_3_)_2_ as described by Ranjit and Klabunde.[^1^](#_ENREF_43) The sharp band at 3740 cm^-1^ for the reference sample is indicative of isolated –OH groups. Since the spectrum for the *in situ*-sample lacks this sharp band, it can be argued that this sample does not contain Mg(OH)_2_. The broad band around 3400 cm^-1^ for both samples are indicative of hydrogen bonded –OH groups, and the sharp bands between 2920 cm^-1^ and 2790 cm^-1^, as well as the band at 1100 cm^-1^, are all indicative of the presence of –OCH_3_ groups. The presence of carbonate groups is clear from the bands between 1650 cm^-1^ and 1440 cm^-1^, while the band around 1030 cm^-1^ is assigned to the *v_C-O_* stretch in the methanol in the sample. The band around 540 cm^-1^ is indicative of the Mg-O bond. Since the spectrum for the *in-situ* sample shares the characteristics of Mg(OH)(OCH_3_) that has been described and well characterised earlier by Ranjit and Klabunde, we believe that one intermediate product in the synthesis of Upsalite is indeed the OHMgOCH_3_ as described in step 1a of the reaction scheme.

**SUPPORTING REFERENCE**

1 Ranjit, K. T.; Klabunde, K. J. Solvent Effects in the Hydrolysis of Magnesium Methoxide, and the Production of Nanocrystalline Magnesium Hydroxide. An Aid in Understanding the Formation of Porous Inorganic Materials. *Chem. Mater.* **2005,** *17*, 65-73.
