# Supplementary material for: A Template-Free, Ultra-Adsorbing, High Surface Area Carbonate Nanostructure
Source: PLoS One. 2013 Jul 17;8(7):e68486. doi: 10.1371/journal.pone.0068486 (PMC3714275; doi:10.1371/journal.pone.0068486)
Supplement: Figure S4 — STEM Tomography and TEM. Internal pore structure from electron tomography. Slices through two electron tomographic reconstructions of the Upsalite material. Essentially representing an internal cross-section, pore size measurements from these volumetric slices indicate pore sizes with one dimension measuring on average 16 nm, and the other varying between 50 nm and 8 nm (S4.1). TEM image showing Upsalite sample after a long period (∼1 min) under the electron beam (S4.2). (DOCX) [file pone.0068486.s004.docx]

SUPPORTING FIGURE S4 for

A template-free, ultra-adsorbing, high surface area carbonate nanostructure

Johan Forsgren, Sara Frykstrand, Kathryn Grandfield, Albert Mihranyan, and Maria Strømme

**S4. STEM Tomography and TEM**

Three-dimensional reconstructions from STEM Tomography allow for visualisation of the internal pore structure of the material via a series of slices through the volume. Measurements from these slices, which essentially represent a cross-section, confirm that pore widths are consistently 16 nm, while pore heights vary between 8 and 50 nm. However, the larger pore networks that are visible with SEM and STEM are not noted throughout the entire material, which is consistent with the limited contribution from these pores to the total pore volume as determined by DFT calculations on nitrogen sorption isotherms.


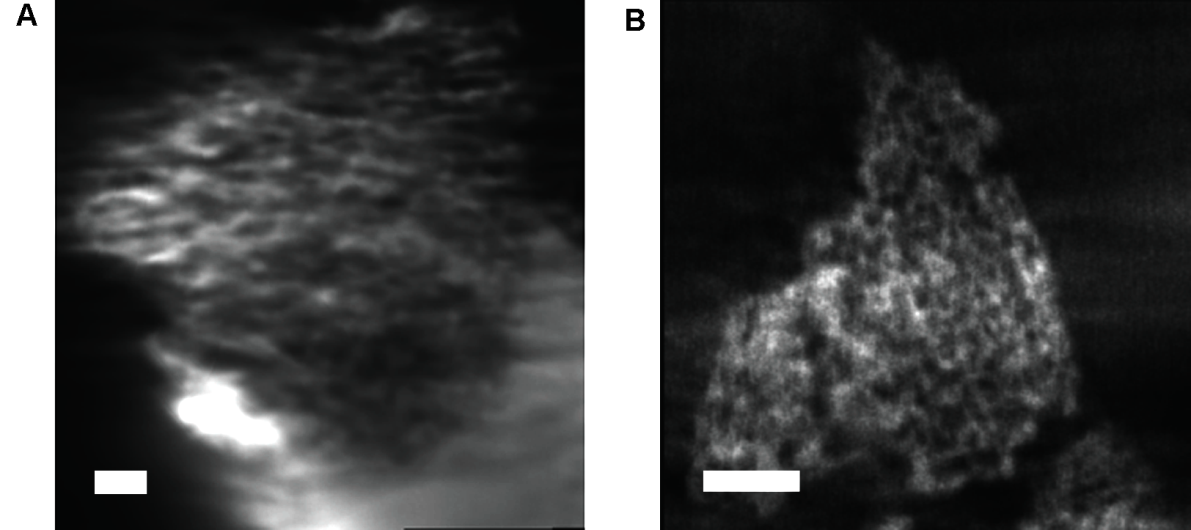


**Figure S4.1.** Internal pore structure from electron tomography. Slices through two electron tomographic reconstructions of the Upsalite material. Essentially representing an internal cross-section, pore size measurements from these volumetric slices indicate pore sizes with one dimension measuring on average 16 nm, and the other varying between 50 nm and 8 nm. Scale bars, 100 nm.


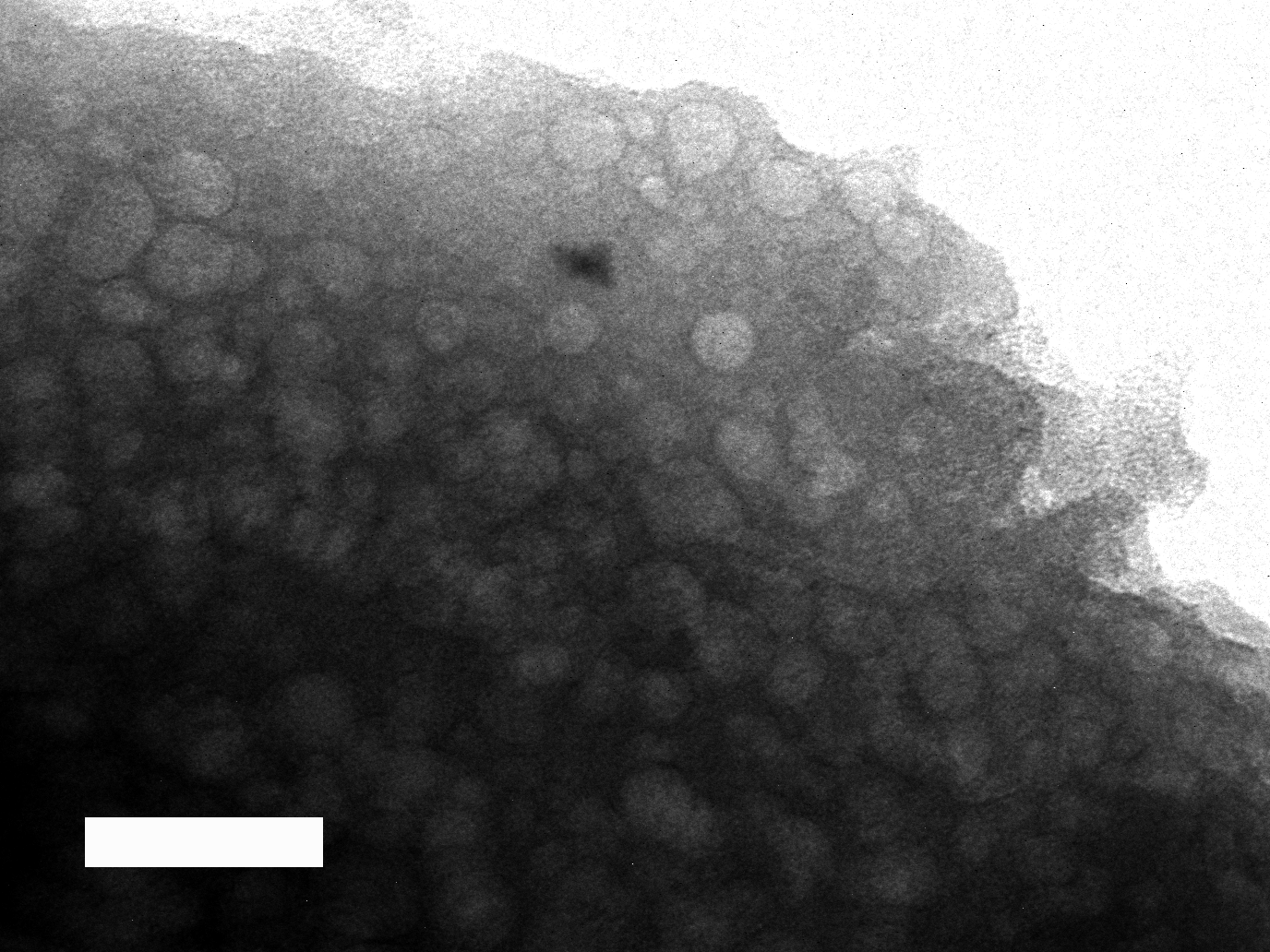


**Figure S4.2.** TEM image showing Upsalite sample after a long period (~1 min) under the electron beam. Scale bar, 50 nm.
