# Supplementary material for: A Template-Free, Ultra-Adsorbing, High Surface Area Carbonate Nanostructure
Source: PLoS One. 2013 Jul 17;8(7):e68486. doi: 10.1371/journal.pone.0068486 (PMC3714275; doi:10.1371/journal.pone.0068486)
Supplement: Figure S5 — Differential Thermal Gravimetric Analysis of Upsalite stored at 100% humidity and RT for different time periods. (DOCX) [file pone.0068486.s005.docx]

SUPPORTING FIGURE S5 for

A template-free, ultra-adsorbing, high surface area carbonate nanostructure

Johan Forsgren, Sara Frykstrand, Kathryn Grandfield, Albert Mihranyan, and Maria Strømme

**S5. TG analysis of Upsalite stored at 100% RH at room temperature**

TG analysis was performed on Upsalite after storage in 100% RH at RT for 11 weeks in order to study the stability of the carbonate. dTGA curves are displayed in Fig. S5. No weakening of the carbonate bond represented by a split of, or a shoulder development on, the peak around 450 °C can be detected. Nor is a shift of this peak towards lower temperatures as compared to the carbonate bond peak of as-synthesised samples, Fig. S2, observed. In fact, the decomposition peak for the carbonate bond is localised at higher temperatures as compared to the as-synthesised material (see Fig. S2), indicating a strengthening of the carbonate bond during the first time periods of storage in moist environments.


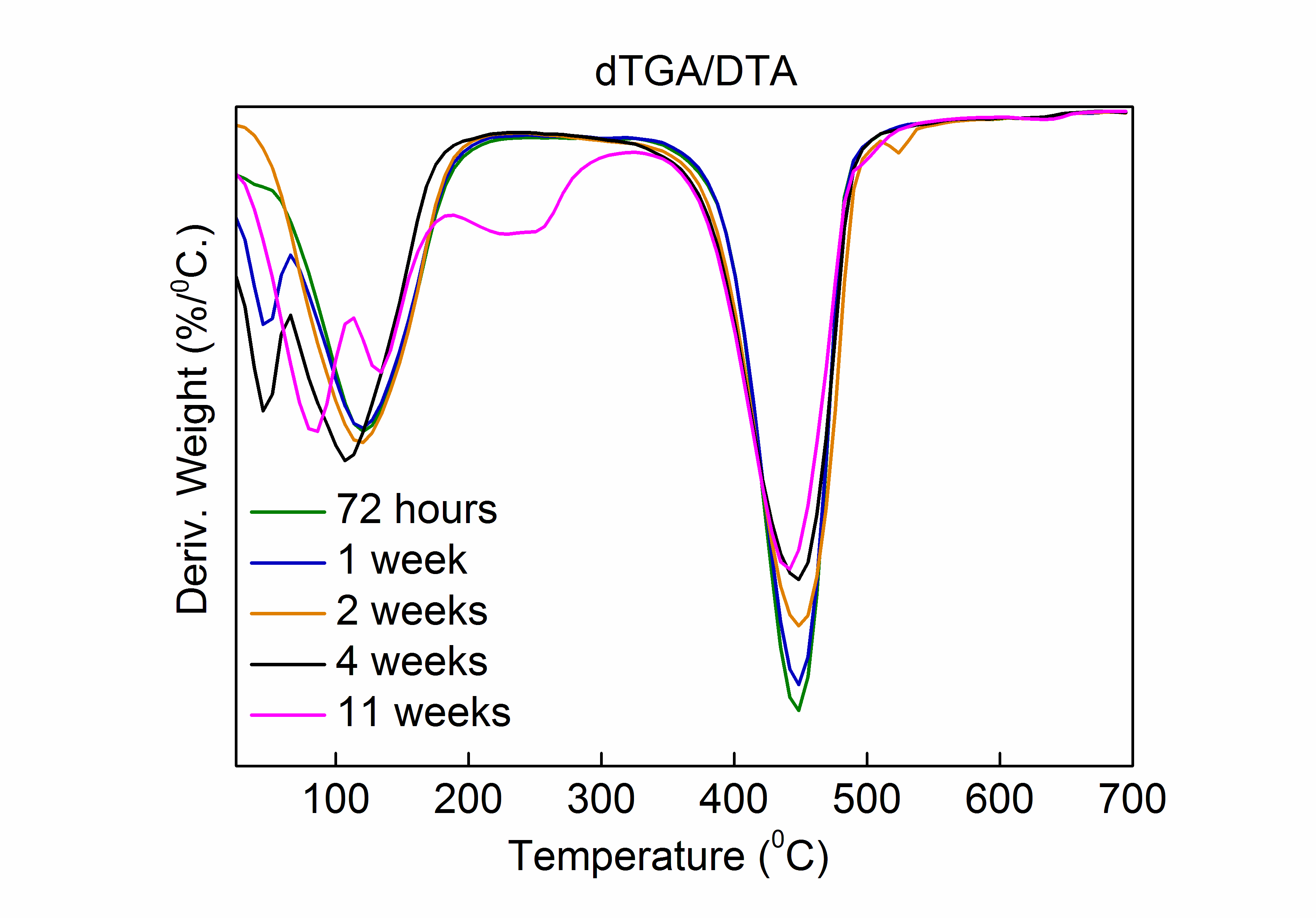


**Figure S5.** Differential Thermal Gravimetric Analysis of Upsalite stored at 100% humidity and RT for different time periods.
