# Supplementary material for: A Template-Free, Ultra-Adsorbing, High Surface Area Carbonate Nanostructure
Source: PLoS One. 2013 Jul 17;8(7):e68486. doi: 10.1371/journal.pone.0068486 (PMC3714275; doi:10.1371/journal.pone.0068486)
Supplement: Text S1 — Overview of earlier work. (DOCX) [file pone.0068486.s006.docx]

SUPPORTING TEXT S1 for

A template-free, ultra-adsorbing, high surface area carbonate nanostructure

Johan Forsgren, Sara Frykstrand, Kathryn Grandfield, Albert Mihranyan, and Maria Strømme

**S1 Overview of earlier work**

For many geologists, the anhydrous magnesite is a conspicuous rock with unclear genesis.[^1^](#_ENREF_1) Although magnesium carbonates are abundant in nature in the form of minor traces in most geological structures, they rarely exist as monomineralic magnesite in economically viable deposits. In fact, there are only two types of magnesite deposits in the world: the sparry magnesite of Vietsch type, which constitutes 90% of world’s reserves and forms nearly monomineralic lenses within marine platform sediments, and the less common but highly valued Kraubath type magnesite of superior quality. The Kraubath type consists of veins (300-400 meter deep) and stockworks (80 meter deep) of cryptocrystalline “bone” magnesite, also sometimes referred to as gel-magnesite.[^1^](#_ENREF_1) It commonly occurs together with ultramafic rock structures such as serpentine ((Mg,Fe)_3_Si_2_O_5_(OH)_4_) and olivine ((Mg,Fe)_2_SiO_4_) minerals, and the formation of Kraubath type magnesite is suggested to occur through a so-called epigenetic-hydrothermal route,[^2-4^](#_ENREF_2) wherein hydrothermal fluids of moderate temperature and low salinity carrying CO_2_ interact with ultramafic rocks. Most of the silica and iron derived from the decomposition of ultramafic rocks are carried to the surface, whereas the veins of magnesite precipitate *in situ* as a gel. It should, however, be stressed that X-ray amorphous forms of MgCO_3_ are not known to occur in nature.

Interestingly, magnesite has aroused problems not only for geologists, but also for chemists. Anhydrous MgCO_3_ can be easily produced at elevated temperatures. However, numerous authors, among them Berzelius,[^5^](#_ENREF_5)^,^ [^6^](#_ENREF_6) Soubeiran,[^7^](#_ENREF_7) Fritzsche,[^8^](#_ENREF_8) Nörgaard,[^9^](#_ENREF_9) Marignac,[^10^](#_ENREF_10) Beckurts,[^11^](#_ENREF_11) Genth & Penfield [^12^](#_ENREF_12), Pfeife [^13^](#_ENREF_13), Knorre [^14^](#_ENREF_14), Doelter, Cornu & Redlich,[^15^](#_ENREF_15) Leitmeier,[^16^](#_ENREF_16) Wells,[^17^](#_ENREF_17) Wilson & Ch'iu,[^18^](#_ENREF_18) and Walter-Lévy^19^ have described unsuccessful attempts to precipitate anhydrous magnesium carbonate from magnesium bicarbonate solutions kept at room temperature and under atmospheric pressure. Instead, hydrated magnesium carbonates or one of the more complex basic magnesium carbonates precipitated under such conditions, leading to what has been branded as *"the magnesite* *problem"*. Significant improvements to produce anhydrous crystalline magnesium carbonate at temperatures below 100 °C were presented in 1999 by Deelman^[20](#_ENREF_20)^ who precipitated magnesite from a suspension of artificial sea-water containing NaCl, MgCl_2_•6H_2_O, MgSO_4_•7H_2_O, KCl, CaCO_3_ and urea at 40 °C. Titration with dilute ammonia together with CO_2_ bubbling through the suspension caused dissolution of the components and precipitation of magnesite after 14 consecutive dissolution-precipitation cycles. However, it should be noted that pure magnesite was not obtained; the precipitates contained both magnesite and different forms of calcium carbonates. This has later been repeated by Dos Anjos et al.[^21^](#_ENREF_21)

For detailed discussion on the different methodologies employed to obtain MgCO_3_, the reader is directed to Deelman.[^22^](#_ENREF_22) For methodologies on the manufacturing of pharmaceutical grades of MgCO_3_, the reader is directed to Truitt.[^23^](#_ENREF_23)

To circumvent *“the magnesite problem”*, some authors have tried to synthesize anhydrous MgCO_3_ in organic solvents and, in particular, in alcohol suspensions since the latter tend to form alkyl esters with alkaline earth metal oxides. The works by the Hungarian chemist Emerich Szarvasy are particularly interesting in this respect. In 1897, Szarvasy has consecutively published 3 articles in *Berichte der Deutschen Chemischen Gesellschaft* (Proceedings of the German Chemical Society) in which he for the first time described several new substances, including magnesium hydroxymethylate (MgOHOCH_3_), magnesium methylate (Mg(OCH_3_)_2_), magnesium dimethylcarbonate (Mg(OCOOCH_3_)_2_), and magnesium dimethylsulfite (Mg(OSOOCH_3_)_2_).[^24-26^](#_ENREF_24) A key substance during Szarvasy’s studies was magnesium methylate, which could be obtained either through direct interaction of magnesium nitride Mg_3_N_2_ or metallic Mg with methanol. Upon interaction with CO_2_ or SO_2_, Mg methylate formed Mg dimethylcarbonate and Mg dimethylsulfite, respectively:[^26^](#_ENREF_26)

CH_3_•O•C•O•O•Mg•O•O•C•O•CH_3_

CH_3_•O•S•O•O•Mg•O•O•S•O•CH_3_

It should also be mentioned that Szarvasy did not describe the monomethyl hydroxycarbonate salt of Mg or its methyl ester;

HO•Mg•O•O•C•O•CH_3_

CH_3_•O•Mg•O•C•O•O•CH_3_

which would otherwise be expected to exist considering the structure of Mg dimethylcarbonate. Magnesium dimethylsulfite could also be obtained by passing sulphur dioxide through the methanolic solution of magnesium dimethyl carbonate by substituting the weak acid ester of carbonic acid by an ester of a stronger acid. As a part of characterisation, Szarvasy also described the following: upon addition of a few droplets of water to magnesium dimethylcarbonate, a white jelly-like precipitate is formed, which can be dissolved in excess water. When the produced clear water solution is boiled, white powder precipitate is formed which is reminiscent of magnesium carbonate. According to Szarvasy, the only water-soluble substance which would precipitate into magnesium carbonate from water upon heating is magnesium hydrocarbonate. Therefore, it was concluded that magnesium dimethylcarbonate should be analogous to magnesium hydrocarbonate, except for the hydroxyl groups being substituted by methoxy groups. Szarvasy also predicted that since magnesium methylate can be produced from direct interaction between metallic Mg and methanol, other alkaline earth metals such as Ca, Sr, and Ba will also form their respective alcoholates.

In 1908, Neuberg and Rewald^[27](#_ENREF_27)^ claimed that the addition of carbonic acid to a methanolic suspension of an alkaline earth metal oxide (e.g. CaO) under mild warming resulted in the dissolution of metal oxide and formation of a colloidal suspension after filtrating away the solid residue. Upon long-standing and moderate stirring in an icebox, the above solution formed a thick starch-like gel, which was rather opaque. The authors stated that the gel was so thick that it could be turned upside down in a glass tube. Upon reaction with acid, such as sulphuric, phosphoric or oxalic acid, this product formed the respective calcium sulphate, phosphate, or oxalate and produces CO_2_ gas, which led the authors to believe that the product may be CaCO_3_. The experiments with MgO, however, turned out differently since when CO_2_ was bubbled through methanolic suspensions of MgO no gel was formed. This led Neuberg and Rewald to doubt that MgCO_3_ can be obtained from methanolic suspensions due to the more likely formation of magnesium dimethyl carbonate,[^27^](#_ENREF_27) as reported earlier by Szarvasy.[^26^](#_ENREF_26) Not so long after, the experiments by Neuberg and Rewald were repeated by Buzágh in 1926.[^28^](#_ENREF_28) Buzágh, another Hungarian chemist, criticised the approach by Neuberg and Rewald as it evidently led to the formation of dimethyl carbonates also for Ca, Ba, and Sr, as it would be anticipated from the teachings of Szarvasy. Then, instead of using oxides, Buzágh suggested to use the respective hydroxides to form pure carbonates of Ca, Ba, and Sr. Yet it remains unclear why Buzágh did not report any data on the reaction of Mg(OH)_2_ with CO_2_ in methanol.

Significant contribution to understanding the properties of dimethylcarbonates of alkali and alkali earth metals was made by Kurov who studied in total 46 different salts of alkyl and alkenyl carbonic acids and their thermal degradation patterns, both with respect to the type of the alcohol used (same metal-different alcohol) and the type of the metal (same alcohol-different metal).[^29-36^](#_ENREF_29) Kurov’s results reveal that in a homologous row of alcohols the most stable salts are dimethylcarbonates and the least stable ones are those of di-n-butylcarbonates; among the monovalent salts the most stable ones are those of Na and the least stable ones are those of Rb; among the divalent salts the most stable ones are those of Ba and least stable ones are those of Mg. The thermal degradation of Mg(OCOOCH_3_)_2_, which was conducted in a sealed ampoule at ~200-300 ˚C, produces MgCO_3_, CO_2_, and CH_3_OH (main components) and traces of (CH_2_)_2_O and CH_2_OH but no CO or C_2_H_4_, as it is the case with monovalent alkali metals.[^32^](#_ENREF_32)

The thermal degradation patterns of mono and di-glycolcarbonic acid salts studied by Franz and Gattow^[37](#_ENREF_37)^ confirmed the general mechanism of thermal degradation proposed by Kurov also for glycolcarbonic salts.

It is postulated in this work that methyl hemicarbonic acid is an important intermediate formed under mild pressure. By pressurising CO_2_, the following reactions are potentially enabled:

ROH+CO_2_↔ HOCOOR (P)

2ROH+CO_2_↔OC(OR)_2_+H_2_O (high P, T, catalysts, desiccants)

In order to form dialkyl carbonates high pressure, high temperature, catalysts (typically metal oxides, including MgO), and desiccants are required.[^38^](#_ENREF_38) The formation of alkylcarbonic acid in CO_2_-alcohol systems has been shown in supercritical fluids at 70-100 bar at 20-40 ˚C for 2 days by interacting it with diazodiphenylmethane as a probe to catch acid species.[^39^](#_ENREF_39) These results also showed that in a homologous row of alcohols the rate of hemicarbonic acid formation is the fastest for methanol and the slowest for tert-butanol. The free methyl hemicarbonic acid was isolated at low temperatures (-70 ˚C) by Gattow and Behrendt.[^40^](#_ENREF_40)^,^ [^41^](#_ENREF_41)

In the current work, FTIR spectroscopy of the reaction products after 3 hours revealed that the main constituent at this stage is HOMgOCH_3_ without traces of Mg(OH)_2_, see Fig. S3 below. It should, however, be noted that magnesium carbonate could also potentially be formed if the product of reaction 1a is Mg methylate rather than Mg hydroxymethylate via formation of Mg dimethylcarbonate as follows:

MgO + 2CH_3_OH ↔ Mg(OCH_3_)_2_+H_2_O (S1a)

CH_3_OH+CO_2_↔CH_3_OCOOH (S1b)

Mg(OCH_3_)_2_+2CH_3_OCOOH↔H_3_CO•CO•O•Mg•O•CO•OCH_3_+2CH_3_OH (S1c)

H_3_CO•CO•O•Mg•O•CO•OCH_3_+H_2_O → MgCO_3_+CH_3_OH +CH_3_OCOOH (S1d)

The last step (S1d) in this reaction actually resembles what Szarvasy described earlier in his work when he adds a few droplets of water to the magnesium dimethylcarbonate as discussed above. In order to verify this hypothesis, Mg(OCH_3_)_2_ was used as the starting material instead of MgO, and water (1:1 mole ratio) was deliberately added to the reaction medium. It was found that indeed an amorphous magnesium carbonate had formed but this material was non-porous and showed a different FTIR spectrum indicating that the short-range order differs from that of Upsalite. Therefore, the reaction scheme 1a-e, i.e. involving HOMgOCH_3_, is more plausible under the employed mild synthesis conditions.

As it is seen from reactions 1d and S1d, the proposed formation of in situ water will induce hydrolysis of the complex methyl ester Mg methylcarbonate. On the other hand, excessive water will halt the formation of hemicarbonic acid through nucleophillic attack as shown below:

CH_3_OCOOH+H_2_O→H_2_CO_3_+CH_3_OH (S1f)

H_2_CO_3_↔ CO_2_+H_2_O (S1g)

Excessive water is also expected to further hydrolyse HOMgOCH_3_ to Mg(OH)_2_. Indeed, we observed that the reaction is extremely sensitive to the presence of water: when water is added deliberately to the methanolic suspension (e.g., 1:1 water/methanol volume ratio) prior to pressurising the reaction vessel, crystalline nesquehonite (Mg(HCO_3_)(OH)•2H_2_O) with no traces of residual MgO is obtained (see Fig. S1).

Another important aspect of the proposed reaction of Upsalite formation is the role of CO_2_ gas pressure. During our synthesis attempts, it became clear that moderately pressurised CO_2_ gas (>1-3 bar) is necessary in order to convert MgO to MgCO_3_ in methanol: when CO_2_ is merely bubbled through a methanolic suspension of MgO, no MgCO_3_ is formed. Reaction 1b is a result of nucleophile attack by methanol on CO_2_, which, due to its intrinsic electron deficiency, can be considered as an anhydride of carbonic acid. The product of the reaction 1b is the methyl ester of hemicarbonic acid and the equilibrium in this reaction is shifted strongly to the left hand-side of the reaction under ambient conditions as discussed above. According to Le Chatelier’s principle, increasing the CO_2_ pressure in reaction 1b will be favourable for shifting the equilibrium in the direction necessary for formation of methyl ester of hemicarbonic acid.

**SUPPORTING REFERENCES**

1. Pohl, W. Genesis of Magnesite Deposits - Models and Trends. *Int. J. Earth. Sci.* **1990,** *79*, 291-299.

2. Braunmühl, H. v. Über Die Entstehung Des Dichten Magnesits Vom Typ Kraubath. *Arch. Lagerstättenforsch.* **1930,** *45*, 1-87, Berlin.

3. Petrascheck, W. E. Zur Bildung Griechischer Magnesitlagerstätten. *Radex-Rundsch.* **1961,** *Heft 4*, 641-646.

4. Dabitzias, S. G. Petrology and Genesis of the Vavados Cryptocrystalline Magnesite Deposits, Chalkidiki Peninsula, Northern Greece. *Econ. Geol.* **1980,** *75*, 1138-1151.

5. Berzelius, J. J. Examen De Quelques Composés Qui Dépendent D’affinités Très-Faibles. *Ann. Chem. Phys.* **1820,** *14*, 363-369.

6. Berzelius, J. J. Untersuchung Über Einige Verbindungen Welche Auf Schwächeren Verwandtschaften Beruhen. *Jour. f. Chemie u. Physik* **1821,** *31*, 258-288.

7. Soubeiran, E. Observations Sur Le Carbonate De Magnésie. *Jour. Pharmacie* **1827,** *13*, 594-601.

8. Fritzsche, J. Ueber Eine Verbindung Der Kohlensauren Talkerde Mit Wasser Und Über Die Magnesia Alba. *Ann. d. Physik* **1836,** *37*.

9. Nörgaard, E. A. Bidrag Til Oplysning Om De Kulsure Magnesiaforbindelser. *Danske Vidensk. Afhandl.* **1851,** *Række 5*, 68-89.

10. Marignac, C. Recherches Sur Les Formes Cristallines De Quelques Composés Chimiques. *Mém. Soc. Phys. Genève* **1855,** *14*.

11. Beckurts, H. Beiträge Zur Kenntnis Der Carbonate Des Magnesiums. *Archiv d. Pharmacie* **1881,** *218*, 429-442.

12. Genth, F. A.; Penfield, S. L. On Lansfordite, Nesquehonite, a New Mineral, and Pseudomorphs of Nesquehonite after Lansfordite. *Am. Jour. Sci.* **1890,** *39*, 121-137.

13. Pfeife, J. Kritische Studien Über Untersuchung Und Reinigung Des Kesselspeisewassers. *Angew. Chem.* **1902,** *15*, 193-207.

14. Knorre, G. V. Über Das Magnesiumkarbonat Und Einige Doppel-Verbindungen Desselben. *Z. Anorg. Chem.* **1903,** *34*, 260-285.

15. Doelter, C.; Cornu, F.; Redlich, K. A. Weitere Vorläufige Mitteilugen Über Arbeiten Auf Dem Grenzgebiete Zwischen Kolloidchemie, Mineralogie Und Geologie. *Colloid Polymer Sci.* **1909,** *4*, 189-190.

16. Leitmeier, H. Über Wasserhaltige Magnesiakarbonate *in* Xxiii. Mitteilungen Der Wiener Mineralogischen Gesellschaft. *Z. Kristallogr. Mineral. Petrol.* **1909,** *28*, 481-486.

17. Wells, R. C. The Solubility of Magnesium Carbonate in Natural Waters. *JACS* **1915,** *37*, 1704-1707.

18. Wilson, E.; Ch'iu, Y. C. Brine Purification - the System Sodium Bicarbonate-Sodium-Chloride-Magnesium Carbonate-Water. *Ind. Eng. Chem.* **1934,** *26*, 1099-1104.

19. Walter-Lévy, L. Carbonates Basiques De MagnéSium. *Annales de Chimie* **1937,** *7*, 1940-1942.

20. Deelman, J. C. Low-Temperature Nucleation of Magnesite and Dolomite. *Neues Jahrbuch Mineral Monatsh* **1999,** *7*, 289-302.

21. Dos Anjos, A. P. A.; Sifeddine, A.; Sanders, C. J.; Patchineelam, S. R. Synthesis of Magnesite at Low Temperature. *Carbonate. Evaporite.* **2011,** *26*, 213-215.

22. Deelman, J. C. In *Low Temperature Formation of Dolomite and Magnesite* [Online] version 2.3; Compact Disc Publications: Eindhoven, 2011. URL: www.jcdeelman.demon.nl (accessed 18 April 2012).

23. Truitt, B. Magnesium Carbonate. In *Handbook of Pharmaceutical Excipients*, 6th ed.; Rowe, R., Sheskey, P., Quinn, M., Eds.; Pharmaceutical Press: London, 2009; pp 397-400.

24. Szarvasy, E. Ueber Das Magnesiummethylat. *Ber. Dtsch. Chem. Ges.* **1897,** *30*, 806-809.

25. Szarvasy, E. Ueber Die Einwirkung Von Methylalkohol Auf Magnesiumnitrid. *Ber. Dtsch. Chem. Ges.* **1897,** *30*, 305-309.

26. Szarvasy, E. Ueber Methylkohlensaures Und Methylschwefligsaures Magnesium. *Ber. Dtsch. Chem. Ges.* **1897,** *30*, 1836-1838.

27. Neuberg, C.; Rewald, B. Ueber Kolloide Und Gelatinöse Verbindungen Der Erdalkalien. *Colloid Polymer Sci.* **1908,** *2*, 354-357.

28. Buzágh, A. Ueber Kolloide Lösungen Der Erdalkalikarbonate. *Kolloid Z.* **1926,** *38*, 222-226.

29. Kurov, V. I. Alkyl Carbonate Salts .1. *Russ. J. Gen. Chem.* **1951,** *21*, 490-493.

30. Kurov, V. I. Alkyl Carbonate Salts .2. *Russ. J. Gen. Chem.* **1951,** *21*, 1637-1642.

31. Kurov, V. I. Alkyl Carbonate Salts. 3. The Use of Tagged Atoms (14C-Isotope) for Studying the Thermal Decomposition of Alkyl Carbonic Salts. *Russ. J. Gen. Chem.* **1957,** *27*, 1754-1755.

32. Kurov, V. I. Alkyl Carbonate Salts .4. *Russ. J. Gen. Chem.* **1959,** *29*, 1245-1249.

33. Kurov, V. I. Alkyl Carbonate Salts .5. Methyl Carbonate Salts of Bivalent Metals. *Russ. J. Gen. Chem.* **1961,** *31*, 9-11.

34. Kurov, V. I. Alkylcarbonic Salts and Their Thermal Degradation. Dissertation thesis, Academy of Sciences of Byelorussian SSR, Minsk, USSR, 1962.

35. Kurov, V. I. Salts of Alkenyl Hydrogen Carbonates .1. *Russ. J. Gen. Chem.* **1967,** *37*, 712-&.

36. Kurov, V. I.; Tsurkan, T. S. Bivalent Salts of Dialkyl Carbonates. *Russ. J. Gen. Chem.* **1967,** *37*, 1146-&.

37. Franz, J.; Gattow, G. Über Chalkogenolate. 77. Untersuchungen Über Glykolkohlensäuren. 2. Glykolmonokohlensäure Und Glykoldikohlensäure. *Z. Anorg. Allg. Chem.* **1977,** *436*, 157-165.

38. Shaikh, A.-A. G.; Sivaram, S. Organic Carbonates. *Chem. Rev.* **1996,** *96*, 951-976.

39. West, K. N.; Wheeler, C.; McCarney, J. P.; Griffith, K. N.; Bush, D.; Liotta, C. L.; Eckert, C. A. In Situ Formation of Alkylcarbonic Acids with Co2. *J. Phys. Chem. A* **2001,** *105*, 3947-3948.

40. Gattow, G.; Behrendt, W. Methyl Hydrogen Carbonate. *Angew. Chem. Int. Ed.* **1972,** *11*, 534-535.

41. Behrendt, W.; Gattow, G. Über Chalkogenolate. Lxii. Untersuchungen Über Halbester Der Kohlensäure 2. Darstellung Und Eigenschaften Der Monomethylkohlensäure. *Z. Anorg. Allg. Chem.* **1973,** *398*, 198-206.
